# Overexpression of programmed cell death ligand 1 in patients with CIN and its correlation with human papillomavirus infection and CIN persistence

**DOI:** 10.1186/s13027-020-00312-9

**Published:** 2020-07-17

**Authors:** Ceyda Sancakli Usta, Eren Altun, Selim Afsar, Cagla Bahar Bulbul, Akin Usta, Ertan Adalı

**Affiliations:** 1 Department of Obstetrics and Gynecology, School of Medicine, Balikesir University, Cagis Yerleskesi, Bigadic yolu 17. km pc:10345, Balikesir, Türkiye; 2 Department of Pathology, School of Medicine, Balikesir University, Balikesir, Turkey; 3 Department of Obstetrics and Gynecology, Balikesir Ataturk State Hospital, Balikesir, Turkey

**Keywords:** Abnormal cytology, Cervical cancer, CIN, Ki-67, PD-L1, p16

## Abstract

**Backround:**

HPV causes specific cell-mediated immunity in the cervix. Mononuclear cells such as helper T cells (CD4+), cytotoxic T cells (CD8+), and dendritic cells play a critical role in the initiation of the HPV-specific immune response and destruction of virus-infected cervical epithelial cells. The programmed cell death ligand 1 (PD-L1) gene encodes an immune inhibitory receptor ligand and overexpression of PD-L1 inhibits T-cell activation and cytokine production. The aim of this study was to investigate the expression of PD-L1 in cervical tissue and its correlation with clinicopathological findings.

**Methods:**

In this cross-sectional study, a total of 94 women who were referred for colposcopy due to abnormal Papanicolaou (PAP) test results and/or HPV positivity were evaluated. The presence of HR-HPV–DNA was analyzed using type- and gene-specific primers along with commercial real-time polymerase chain reaction. The cervical examination was done with a colposcope. Cervical biopsies were obtained from the areas that were evaluated as abnormal during the colposcopy. Histopathological result of cervical biopsies were defined as no intraepithelial neoplasia (CIN 0), mild CIN (CIN I), and moderate-to-high CIN (CIN II-III). All women were classified into four groups based on their HR-HPV positivity and cervical biopsy results: Group I (controls; *n* = 29), HR-HPV (−) CIN 0; Group II (*n* = 21), HR-HPV (+) CIN 0; Group III (*n* = 20), HR-HPV (+) CIN I; and Group IV (*n* = 24), HR-HPV (+) CIN II-III. A semi-quantitative scoring system was used to evaluate the degree of Ki-67, p16, and PD-L1 immunoreactivity in the cervical tissue samples.

**Results:**

We found that PD-L1 expression in both mononuclear cells and in cervical epithelial cells gradually increases from the HR-HPV (−), CIN 0 group to the HR-HPV (+), CIN II-III group (*p* = 0.0003 and *p* = 0.0394, respectively) and mononuclear PD-L1 expression was correlated with HPV type, initial Pap test results, HPV persistence, and CIN persistence or recurrence (*p* = 0.0180, *p* = 0.0109, *p* = 0.0042, and *p* = 0.0189, respectively). Moreover, mononuclear PD-L1 expression was also correlated with Ki-67 and p16 immunoreactivity (*p* = 0.0432 and *p* = 0.0166, respectively). Epithelial PD-L1 expression was only correlated with HPV type and the presence of HPV persistence (*p* = 0.0122 and *p* = 0.0292, respectively).

**Conclusion:**

During the initial evaluation of the cervical histology results, the assessment of PD-L1 expression—especially in mononuclear cells in cervical tissue samples—may provide more information on the progression of HR-HPV infection and its persistence.

## Background

Cervical cancer is one of the most common gynecological malignancies affecting women worldwide. The incidence and mortality rates vary depending on the use of effective screening strategy, sexual behavior, race, and socioeconomic status, and are approximately 7.8 and 2.3 per 100,000, respectively in the United States [1]. It is estimated that squamous cell carcinoma (SCC) accounts for 90% of all Cervical cancers [2]. Human papilloma virus (HPV) infection in the cervical epithelium is the main etiological factor for the development of SCC [3]. The presence of persistent infection with a high malignancy potential HPV causes cervical intraepithelial neoplasia (CIN) and, subsequently, SCC.

HPV infection in the cervix causes HPV-specific cell-mediated immunity that plays an important role in the clearance or persistence of HPV infection [4, 5]. During the HPV infection, helper T cells (CD4+), cytotoxic T cells (CD8+), and dendritic cells play a critical role in the initiation of the HPV-specific immune response and destruction of virus-infected cervical epithelial cells. The integration of the high-risk HPV (HR-HPV) genome into the epithelial cells of the cervix results in the abnormal regulation of the cell cycle controls [6]. Dendritic cells are the most potent antigen-presenting cells for naive T cells. The presentation of viral antigens by dendritic cells activates naive T cells to proliferate and differentiate into effector cells, which produce cytokines that amplify the antiviral response or specifically recognize and eliminate the virus-infected cells [7, 8].

It is also known that HPV is capable of immune evasion (immune escape) that prevents a robust immune response from the infected cells [6]. HPV-mediated immune system modifications include tumor-associated macrophage differentiation, a compromised cellular immune response, an abnormal imbalance between helper T cells (CD4+) and cytotoxic T cells (CD8+), regulatory T cell infiltration, and downregulated dendritic cells activation and maturation [6]. Thus, HPV can remain undetectable for a long time [6]. However, the molecular and cellular mechanisms underlying the clearance or persistence of cervical HPV infection are still largely unknown.

The Programmed cell death ligand 1 (PD-L1 or CD274) gene encodes an immune inhibitory receptor ligand that is expressed by hematopoietic and non-hematopoietic cells, such as T cells, B cells, and various types of tumor cells [9]. The interaction of PD-L1 with its receptor inhibits T-cell activation and cytokine production [10, 11]. Related studies have shown that this pathway plays a critical role in attenuating T-cell activation and promoting T-cell tolerance during infection with human immunodeficiency virus, hepatitis B virus, hepatitis C virus, and other pathogens capable of establishing chronic infections [12, 13]. Although considerable evidence supports the involvement of PD-L1 expression in the negative regulation of many adaptive responses, it is not yet known whether it influences the human anti-HPV response or contributes to HPV immune evasion [12, 13].

Therefore, we aimed to investigate the roles of PD-L1 expression in the pathogenesis of HR-HPV–related CIN. Thus, we examined the expression of PD-L1 on the mononuclear cells and cervical epithelial cells of cervical tissue samples from HR-HPV (−) CIN 0 and HR-HPV positive (+) women with different CIN grades and the correlation with clinicopathological findings.

## Methods

### Ethical statement

The investigation protocol of this study was in accordance with the Helsinki Committee requirements. All participant data were obtained from Balikesir University, School of Medicine, Department of Gynecology and Obstetrics between January 2015 and April 2020. Ethical approval was given by the institutional Ethical Committee of Balikesir University, School of Medicine (2020/64). Written informed consent was obtained from all participants.

### Patients’ characteristics

In this cross-sectional study, a total of 94 women who were referred for colposcopy due to abnormal Papanicolaou (PAP) test results and/or HPV positivity in the co-test were included in the study population. All participants had an abnormal Papanicolaou (Pap) test results that included atypical squamous cells of undetermined significance (ASCUS), low-grade cervical intraepithelial lesion (LSIL), high-grade cervical intraepithelial lesion (HSIL), atypical squamous cells-cannot exclude high-grade squamous intraepithelial lesion (ASC-H), and/or HPV infection. All women were examined by colposcopy at the next visit. Cervical biopsies were obtained during the colposcopic examinations. Cervical biopsy results were defined as no intraepithelial neoplasia (CIN 0), mild CIN (CIN I), and moderate-to-high CIN (CIN II-III) based on a consensus review by two experienced pathologists. All women were classified into four groups based on their HR-HPV and cervical biopsy results: Group I (controls; *n* = 29), HR-HPV (−) CIN 0; Group II (*n* = 21), HR-HPV (+) CIN 0; Group III (*n* = 20), HR-HPV (+) CIN I; and Group IV (*n* = 24), HR-HPV (+) CIN II-III (Table 1).

**Table 1:** Clinical and pathological results of patients in the groups

	Group 1 (***n*** = 29) HR-HPV(−) CIN 0	Group 2 (***n*** = 21) HR-HPV(+) CIN 0	Group 3 (***n*** = 20) HR-HPV(+) CIN1	Group 4 (***n*** = 24) HR-HPV(+) CIN2–3	***P*** value
**Age (year), mean ± SD**	44.3 ± 10.6	42.2 ± 12.4	42.7 ± 10.3	39.1 ± 7.6	0.1698*
**Parity, n (min-max)**	1 (0–4)	2 (0–6)	1 (1–5)	2 (0–5)	0.1245^#^
**Initial pap test results**					
**ASCUS**	22	7	4	1	**< 0.0001**^**#**^
**L-SIL**	7	9	11	5	
**H-SIL or ASC-H**	–	5	5	18	
**HR-HPV types**					
**16**	–	9	4	3	
**18**	–	8	8	5	**< 0.0001**^**#**^
**Other**	–	0	5	5	
**Multiple**	–	4	3	11	
**Ki-67, median (min-max)**	25 (5–60)	29 (0–85)	24 (5–100)	82 (30–100)	**< 0.0001**^**$**^
**p16, median (min-max)**	25 (0–80)	34 (0–51.5)	20 (0–66)	67,5 (15–100)	**0.0025**^**$**^
**Number of immune cells**	27.9 ± 6.7	33.5 ± 6.2	34.1 ± 5.8	36.3 ± 6.9	0.4190*
**Leep or Conization rate, n (%)**	0	1/21	8/20	24/24	**< 0.0001**^**#**^
**HPV persistence**	–	2	3	4	**0.0296**^**#**^
**CIN persistence or recurrence**	–	1	0	3	0.0581^#^

*ANOVA, ^#^Chi-Squared, ^$^Kruskal Wallis test

Ki-67 expression: Goup IV different from group I, II, III. No differences between group I, II, III

p16 expression: Group IV different from group I, II, III. No differences between group I, II, III

All procedures were performed according to current guidelines of the American Society for Colposcopy and Cervical Pathology [14]. Patients who had cervical infections with other agents, had immunological diseases, used anti-inflammatory and immunosuppressant drugs, had sexually transmitted diseases and neoplasia, or were pregnant were excluded from the study.

### Detection and typing of HPV

For the collection of the cervical samples, a cervical cyto-brush and 1 mL commercial transport medium (QIAGEN, Venlo, Netherlands) was used. HPV-DNA was extracted from cervical samples using a DNeasy Blood & Tissue Kit (QIAGEN) according to the manufacturer’s instructions. Detection of HR-HPV–DNA was performed using type- and gene-specific primers along with commercial real-time polymerase chain reaction (Rotor Gene Q, QIAGEN). The presence of the HPV types 16, 18, 31, 33, 35, 39, 45, 51, 52, 56, 58, 59, and 66, was analyzed by polymerase chain reaction (PCR) test as described elsewhere [15].

### Cervical cytological evaluation

Cervical cytological samples were taken with a cyto-brush and a conventional Pap test was used for the detection of cervical cytological abnormalities. Cytological diagnoses were obtained using the 2001 Bethesda System [16]. For cytological evaluation, we evaluated the presence of atypia, the degree of atypia, cell irregularity, increased nuclear-cytoplasmic ratio and koilocytosis.

### Cervical histology specimen examination and immunohistochemistry procedures

During the colposcopy, cervical samples were taken from the areas that were evaluated as abnormal with punch biopsy forceps. All cervical specimens were fixed with formalin and embedded in paraffin. Sections that were 4-μm thick were cut and stained with hematoxylin and eosin. For the histological examination, we evaluated the presence of cell dysplasia, cell irregularity, cell hyperchromatism, and increased nuclear-cytoplasmic ratio, loss of cell polarization, mitotic figure, koilocytosis, and infiltration of the immune cells.

All tissue preparation and immunohistochemistry (IHC) procedures were performed as previously described [17]. IHC analysis was performed using the Super Vision Assay Kit (SV0002–1, Boster Bio, Pleasanton, CA, USA) by two experienced histopathologists. The primary antibodies used in this study were an anti-human Ki-67 antibody against proliferating cells, dilution 1:100 (Anti-human Ki-67 Antigen Antibody, Dako, Carpinteria, CA, USA), an anti-mouse p16 (p16INK4a) monoclonal antibody, dilution 1:50 (Roche E6H4™, catalog #725–4713, Roche, Basel, Switzerland), and an anti-mouse Anti-PD-L1 antibody, dilution 1:100 [PDL1/2746] (ab238697, Abcam, Cambridge, MA, USA). During the evaluation, analyzed fields were randomly selected from ten slides using a light microscope (Nikon Eclipse Ni-U, Tokyo, Japan).

### Evaluation of the IHC results

**Fig. 1:**
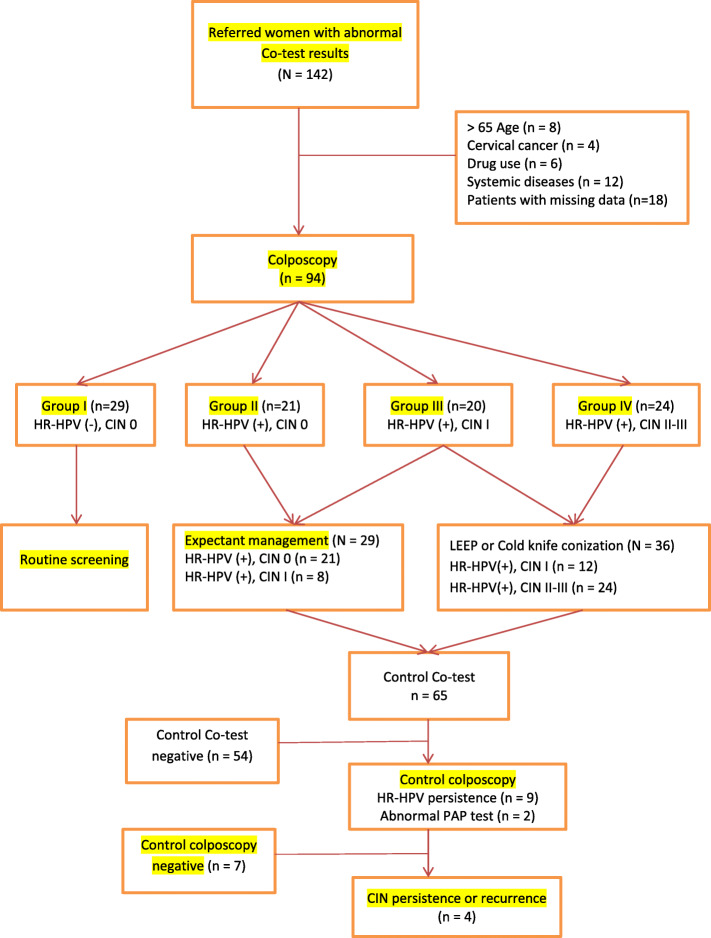
Differential expression of PD-L1 in cervical tissues by immunohistochemistry (× 200). **a**, Weak expression of PD-L1 in a woman without CIN (light brown staining, score 1). **b**, PD-L1 expression in a woman with CIN III revealing strong membranous staining in cervical epithelial cells and infiltrating mononuclear cells (dark brown, score 3). e, epithelium; s, stroma

**Fig. 2:**
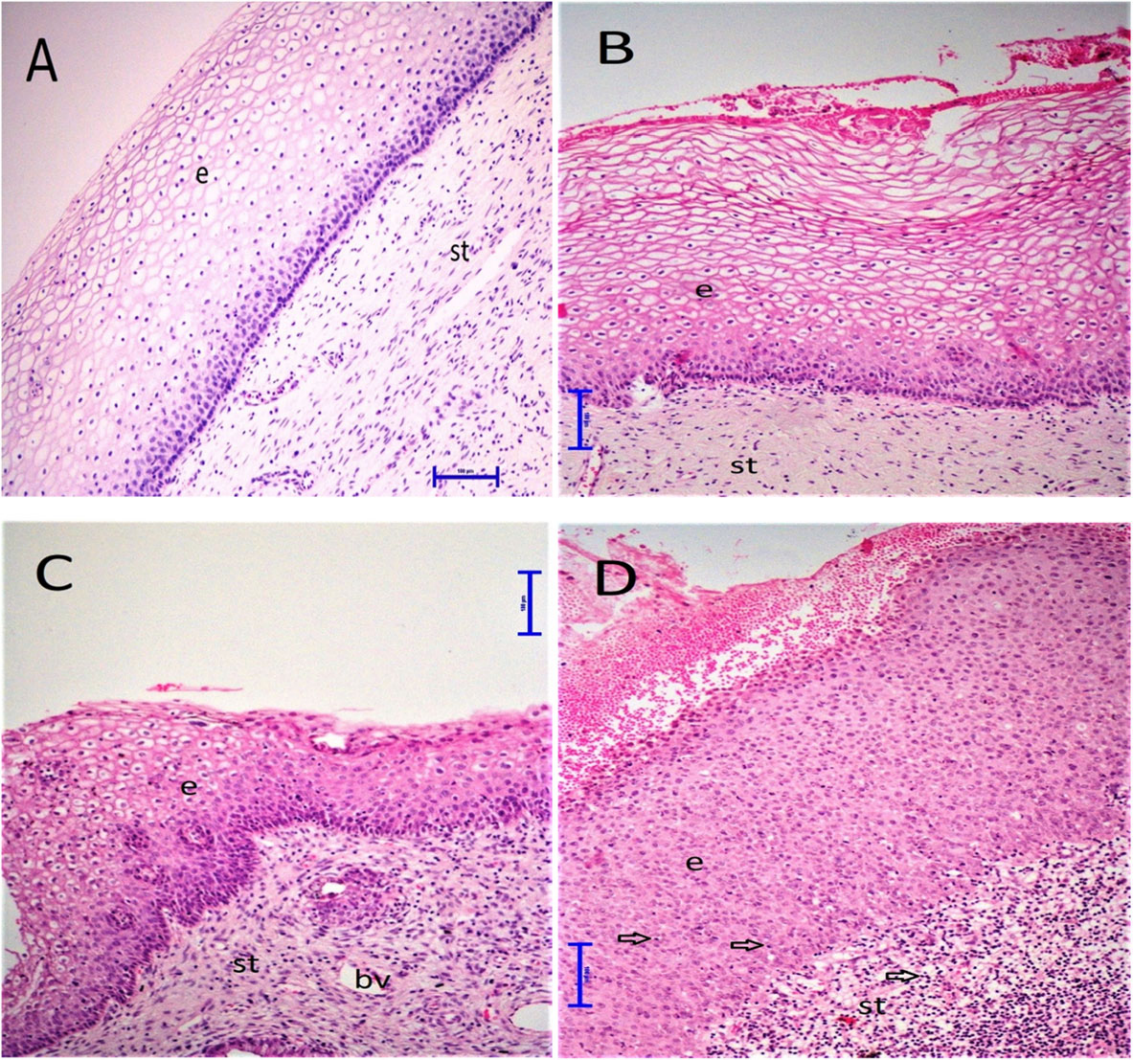
Differential expression of Ki-67 in cervical tissues by immunohistochemistry (× 200). **a**, Weak expression of Ki-67 in a woman with CIN I. **b**, Ki-67 expression in a woman with CIN III revealing strong nuclear staining in cervical epithelial cells. e, epithelium; s, stroma

**Fig. 3:**
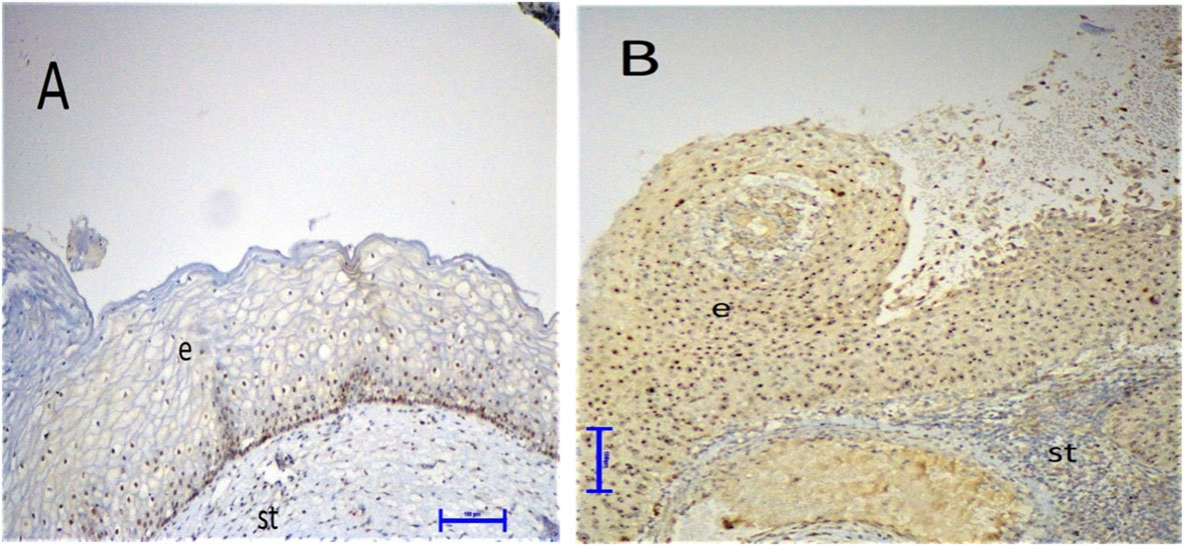
Differential expression of p16 in cervical tissues by immunohistochemistry (× 200). **a**, Weak expression of p16 in a woman with CIN I. **b**, p16 expression in a woman with CIN III revealing strong nuclear and cytoplasmic staining in cervical epithelial cells. e, epithelium; s, stroma

In all patients, the cervical tissue samples showed only moderate immunoreactivity for p16 and PD-L1, but showed strong immunoreactivity for Ki-67. The Ki-67 expression was predominantly localized in the nucleus of the cervical cells. The p16 expression was predominantly localized in the nucleus and cytoplasm of the cervical cells. Based on this observation, cytoplasmic p16 immunoreactivity alone was considered as negative. The PD-L1 expression was predominantly localized in the cell membrane of both cervical and mononuclear cells. Thus, we evaluated PD-L1 expression in these cells separately. All samples were stained with Ki-67, p16, and PD-L1 at the same time. During the evaluation, two experienced histopathologists were blinded to the clinical diagnosis and the cytological results of the patients (Figs. 1, 2 and 3).

A semi-quantitative scoring system was used to evaluate the degree of Ki-67, p16, and PD-L1 immunoreactivity. The degree of positive staining for Ki-67 was calculated by counting 100 endometrial epithelial cells: < 5 cells with nuclear staining were evaluated as negative; 6–25 cells as 1+; 26–50 cells as 2+; 51–75 as 3+; and ≥ 76 as 4+. The p16 immunoreactivity was evaluated based on four parameters: (1) intensity: strong (dark brown color similar to the positive control) versus weak (yellow color significantly lighter than the positive control); (2) extent: diffuse (signal involves > 50% of the epithelium) versus focal (< 50% of the epithelium); (3) continuity: continuous (staining extends laterally over a significant distance) versus discontinuous (alternating clusters of either positively or negatively stained cells); and (4) location: positive cells reside in the lower third, two thirds, or full thickness of epithelium. Based on these four parameters, lesions were categorized as negative (0), ambiguous pattern (1+), and block-positive (2+). PD-L1 immunoreactivity was categorized based on the percentage of membranous positivity of the cells: < 5% positive cells per field as 0; 5–29% positive cells per field as 1+; 30–59% positive cells per field as 2+; and ≥ 60% positive cells per field as 3 + .

### Statistical analysis

All statistical analyses were performed using MedCalc Statistical Software version 19.2.1 (MedCalc Software Ltd., Ostend, Belgium; https://www.medcalc.org; 2020). The distribution of all variables in both the CIN (+) and control groups were studied by describing the mean ± standard deviation (SD) or median (min-max), where applicable. Whether the distributions of continuous variables were normal or not was determined by Kolmogorov–Smirnov test. Also, the Levene’s test or F test was used for the evaluation of the variances. The Student’s t-test was used to compare normally distributed measurements for independent samples and the Mann–Whitney U test was applied for comparisons of the median values. The Chi-square test was used to compare categorical data. While the mean differences among more than two independent groups were analyzed by one-way analysis of variance (ANOVA), the Kruskal–Wallis test was applied for comparisons of the median values. When the *p*-value from one-way ANOVA or Kruskal–Wallis test statistics was statistically significant, the Scheffé test or post-hoc analysis nonparametric multiple comparison test was used to determine which group differed from which. A *p*-value of < .05 was considered statistically significant.

## Results

In this cross-sectional study, the cervical sitology and histology results of 94 patients were evaluated. The mean ages and median parity of the patients were similar between the groups (*p* = 0.1698 and *p* = 0.1245, respectively). Clinical and pathological characteristics of the patients were summarized in Table 1.

### Cytological and histopathologic diagnosis and CIN grading

**Fig. 4:**
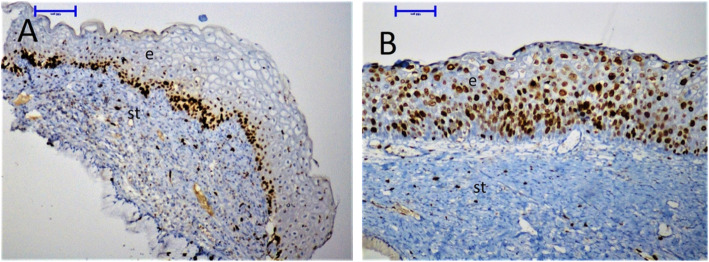
Hematoxylin–Eosin staining in cervical tissue sample (× 200). **a**, normal cervical tissue. The cervical tissue were mainly composed of stromal tissue (fibrous connective tissue), blood vessels, and non-keratinized stratified squamous epithelium. **b**, Cervical tissue samples from women with CIN I. The nuclei of the dysplastic cells were enlarged, irregular, and hyperchromatic, and the histopathologic evaluation of these patients demonstrated a slight dysplasia limited to the basal third of the cervical epithelial layer. **c** and **d**, Cervical tissue samples from women with CIN II and III. Moderate or severe dysplasia and more abnormal cells were detected in at least two-thirds of the epithelial layer of the cervical samples in CIN II or III patients. There was an increased nuclear-to-cytoplasmic ratio, loss of cell polarization, and mitotic figures present in dysplastic cells of CIN II-III patients. e, epithelium; s, stroma; bv, blood vessels; arrow, mononuclear cells

The morphological analysis of cervical specimens by light microscopy showed that there was no morphological difference in cervical cytological and histopathologic samples in CIN 0 patients. However, in CIN I patients, the nuclei of the dysplastic cells were enlarged, irregular, and hyperchromatic, and the histopathologic evaluation of these patients demonstrated a slight dysplasia limited to the basal third of the cervical epithelial layer. Also, moderate or severe dysplasia and more abnormal cells were detected in at least two-thirds of the epithelial layer of the cervical samples in CIN II or III patients. There was an increased nuclear-to-cytoplasmic ratio, loss of cell polarization, and mitotic figures present in dysplastic cells of CIN II-III patients. Extension into the endocervical glands was common, but no invasive SCCs were diagnosed. Samples from the HR-HPV (+) groups frequently showed koilocytosis of the epidermal prickle cell layer and increased numbers of infiltrating immune cells (Fig. 4a-d).

### Presence of HR-HPV persistence and CIN persistence or recurrence

**Fig. 5:**
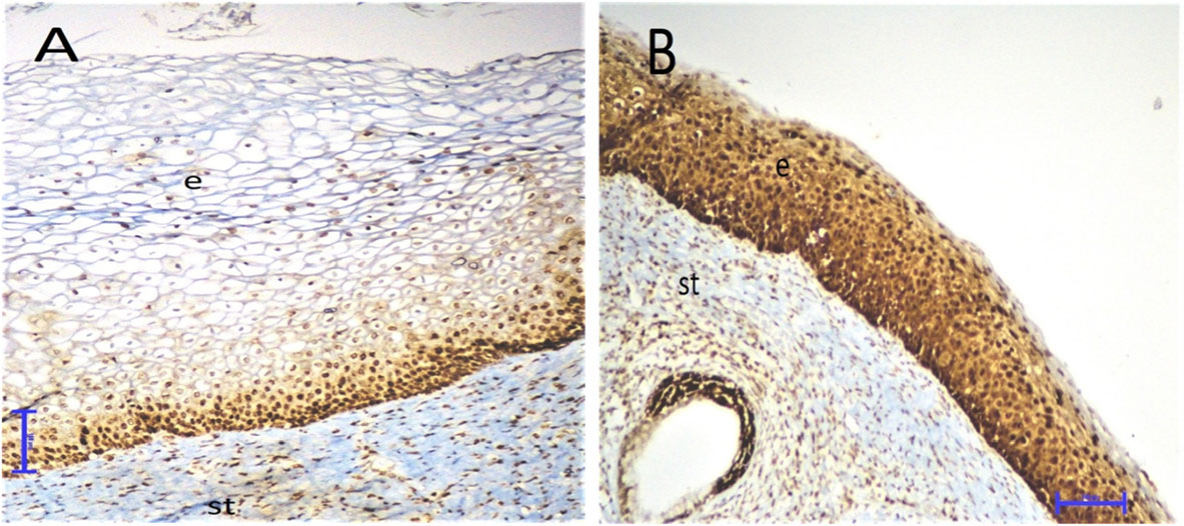
Flow chart showing recruitment of the study population and management of abnormal HPV-DNA and PAP test results

According to control co-tests and subsequent colposcopy results of Group II-IV women, 2/21 in Group II, 3/20 in Group III, and 4/24 in Group IV had HPV persistence, and 1/2 in Group II, 0/3 in Group III, and 3/4 in Group IV women had CIN persistence or recurrence. Finally, a total 33 of 94 women underwent LEEP or cold-knife conization (1/21 in Group II, 8/20 in Group III, and 24/24 in Group IV) (Fig. 5).

### Increased expression of PD-L1 on mononuclear and cervical epithelial cells in HR-HPV–related CIN

Importantly, we have shown that PD-L1 expression in both mononuclear and cervical epithelial cells gradually increases from the HR-HPV (−), CIN 0 group to the HR-HPV (+), CIN II-III group and there was a statistically significant difference between the groups (*p* = 0.0003 and *p* = 0.0394, respectively). Our subgroup analysis demonstrated that the HR-HPV (+), CIN II-III group revealed a significantly higher PD-L1 expression in mononuclear cells than the HR-HPV (+), CIN 0 (*p* = 0.0034) and HR-HPV (−), CIN 0 groups (*p* = 0.0016). Moreover, the PD-L1 expression of mononuclear cells was significantly higher in the HR-HPV (+), CIN 0 group than in the HR-HPV (−), CIN 0 group (*p* = 0.0477). However, there was no difference in mononuclear PD-L1 expression in the CIN I and CIN II-III (*p* = 0.3827) groups. We also found that epithelial PD-L1 expression in the HR-HPV (+), CIN II-III group was significantly higher than in the HR-HPV (−) CIN 0 (*p* = 0.0154) and HR-HPV (+) CIN 0 (*p* = 0.0130) groups (Table 2 and Fig. 1a, b).

**Table 2:** Mononuclear and epithelial PD-L1 expressions in cervical tissue specimens of patients in the groups

PD-L1 Staining Score		Group 1 (***n*** = 29) HR-HPV(−), CIN 0	Group 2 (***n*** = 21) HR-HPV(+), CIN 0	Group 3 (***n*** = 20) HR-HPV(+), CIN 1	Group 4 (***n*** = 24) HR-HPV(+), CIN 2–3	***P*** value^**#**^
Mononuclear PD-L1 expression, n (%)	0	13	3	1	2	0.0003
	1	9	10	7	4	
	2	5	8	7	9	
	3	2	0	5	9	
Epithelial PD-L1 Expression, n (%)	0	22	14	15	9	0.0394
	1	4	6	1	8	
	2	3	1	2	3	
	3	0	0	2	4	

^#^Chi-Squared test

Mononuclear PD-L1 expression: Grup I vs II: 0.0477, group I vs III; 0.0125, group I vs IV: 0.0016, group II vs III: 0.0302, group II vs IV: 0.0034, group II vs IV: 0.3827

Epithelial PD-L1 expression: Group I vs II: 0.3776, group I vs III: 0.2840, group I vs IV: 0.0154, group II vs III: 0.1156, group II vs IV: 0.0130, group III vs IV: 0.0573

In accordance with these results, the correlation analysis showed that the mononuclear PD-L1 expression was correlated with clinicopathological parameters including HPV type, initial Pap test results, HPV persistence, and CIN persistence or recurrence after the initial evaluation (*p* = 0.0180, *p* = 0.0109, *p* = 0.0137, and *p* = 0.0308, respectively). Whereas, cervical epithelial PD-L1 expression was only correlated with HPV type and the presence of HPV persistence (*p* = 0.0122 and *p* = 0.0292, respectively) (Table 3).

**Table 3:** Correlation analysis between PD-L1 expressions in mononuclear and epithelial cells with clinopathological variables of patients

	Mononuclear PD-L1 expression	Epithelial PD-L1 expression
Age (year)	0.01117	0.1107
	*p* = 0.9149	*p* = 0.2881
**HPV types**	**0.2436**	**0.2576**
	***p*** **= 0.0180**	***p*** **= 0.0122**
**Initial pap test results**	**0.2614**	0.1619
	***p*** **= 0.0109**	*p* = 0.1190
Number of immune cells	0.1189	0.1348
	*p* = 0.3457	*p* = 0.1953
**HR-HPV persistence**	**0.2534**	**0.2250**
	***p*** **= 0.0137**	***p*** **= 0.0292**
**CIN persistence or recurrence**	**0.2229**	0.1198
	***p*** **= 0.0308**	*p* = 0.2499
**Ki-67 expression**	**0.2105**	0.1102
	***p*** **= 0.0417**	*p* = 0.2537
**P-16 expression**	**0.2686**	0.1905
	***p*** **= 0.0088**	*p* = 0.0659

Also, Ki-67 and p16 expression was compared between the groups per randomly selected fields of the tissue samples by light microscopy. Importantly, we found that HR-HPV (+) CIN II-III women had significantly increased Ki-67 and p16 expression in their cervical tissue samples than HR-HPV (−) women and HR-HPV (+) women with CIN 0 or I (*p* <  0.0001 and *p* = 0.0025, respectively). There were no differences between HR-HPV (−) women and HR-HPV (+) women with CIN 0 and I (*p* > 0.05). We also evaluated the correlation of mononuclear and cervical epithelial PD-L1 expression with Ki-67 and p16 staining and there was a positive correlation (*p* = 0.0417 and *P* = 0.0088, respectively) (Figs. 2 and 3).

## Discussion

In this cross-sectional study, we evaluated the expression of PD-L1 in the mononuclear and cervical epithelial cells of women who were HR-HPV (−) CIN 0 and HR-HPV (+) with CIN 0, I, and II-III. According to our results, HR-HPV (+) women with CIN 0, I, and II-III demonstrated significantly higher PD-L1 immunoreactivity in their mononuclear and cervical epithelial cells compared to HR-HPV (−) CIN 0 group. Moreover, PD-L1 expression was correlated with the presence of HR-HPV infection, HPV persistence, and CIN persistence or recurrence. Additionally, there was a positive correlation between mononuclear PD-L1 expression and Ki-67 and p16 staining in the cervical tissue samples. To our knowledge, this is the first study to investigate the expression of PD-L1 and its relationship with the histopathological changes of the cervical tissue of women with CIN.

In the literature, a number of studies have demonstrated that the immunohistochemical expressions or circulating levels of PD-L1 are significantly higher in many types of cancers including lung, gastric, prostate, ureteral, and cervical [18–25]. Moreover, PD-L1 expression is associated with an advanced grade of cancer, presence of metastasis, poor prognosis, resistance to chemotherapy, and increased mortality [15, 18–25]. Karpathiou et al. found that immune cell and tissue expression of PD-L1 were higher in SCC patients with lymph node metastasis and that the overexpression of PD-L1 is associated with a poor prognosis [20]. Zhang et al. demonstrated an increased expression of PD-L1 in patients with upper urogenital tract carcinoma and that PD-L1 expression is an independent predictor of decreased cancer survival [24]. In a recent review report by Wang et al., the PD-L1 expression of different human cancers and its associations with clinical outcomes were investigated and it was concluded that PD-L1 is involved in the pathogenesis and progression of numerous malignancies [22].

On the other hand, there are few studies evaluating PD-L1 expression in patients with CIN [15, 26]. Yang et al. demonstrated that PD-L1 expression in cervical T cells and DCs was significantly higher in patients with HR-HPV positivity and gradually increased in correlated with an increasing CIN grade. The authors concluded that the up-regulation of PD-L1 expression may negatively regulate cervical cell–mediated immunity to HPV and contribute to the progression of HR-HPV–related CIN [15]. Mezache et al. found that the presence of HPV infection in the cervix was strongly associated with PD-L1 expression, which was found in both the dysplastic and neoplastic squamous cells and adjoining cytotoxic T (CD8+) cells. Moreover, they demonstrated that PD-L1 expression may be able to differentiate productive HPV infection, which is typically treated with ablative therapy. This finding shows us that a productive viral infection is necessary to induce PD-L1 expression [26]. Comparable with these results, we have shown that both mononuclear and epithelial PD-L1 expression gradually increased from HR-HPV (−) to HR-HPV (+) with CIN II-III, and there was a significant correlation between mononuclear PD-L1 expression with the presence of HR-HPV and abnormal Pap test results. Moreover, we also showed that mononuclear PD-L1 expression correlated with the persistence of HR-HPV infection and the persistence or recurrence of CIN. All of these results indicated that PD-L1 may be associated with the initiation and progression of cervical cell dysplasia in the presence of HR-HPV infection and its persistence. However, PD-L1 expression between women with CIN I and CIN II-III did not reach statistical significance in the present study. This result may be associated with the small number of patients that were included in the study population. We strongly recommend a further study to test whether or not the differences of PD-L1 expression between CIN I and CIN II-III patients can be confirmed in a larger study population.

It is known that Ki-67 is a proliferating cell marker, and previous studies have demonstrated that Ki-67 is a sensitive biological indicator of CIN progression [27]. The p16 protein is a cyclin-dependent kinase inhibitor that acts as a G1-S transition checkpoint of the cell cycle [28]. Previous studies clearly demonstrated that the increased expression of these biomarkers has been associated with the severity and progression of cervical neoplasia [29–33]. Recent studies also showed that there was a correlation of the overexpression of these biomarkers with a worse prognosis. In a systematic review with meta-analysis conducted by Piri et al., it was clearly demonstrated that Ki-67 expression is more predictive than standard histopathological grading of CIN progression [33]. Kava et al. showed that p16 is a robust, stable, and strong predictive biomarker of CIN prognosis [29]. In the present study, we found that HR-HPV (+) women with CIN II-III had significantly higher Ki-67 and p16 expressions in the cervical tissue samples than those of HR-HPV (−) women and HR-HPV (+) women with CIN 0 or I. Our present results indicated that the overexpression of Ki-67 and p16 indicates the presence of advanced cervical lesions rather than HR-HPV positivity. This result may be associated with the patient characteristics of the studied population. We also found that there was a positive correlation between mononuclear PD-L1 expression and Ki-67 and p16 expressions. These results have shown that as the viral load increases, the mitotic rate and PD-L1 expressions increase, which means that immunotherapeutic approaches can differ across different stages of HPV infection.

## Conclusions

In conclusion, PD-L1 expression in both mononuclear and cervical epithelial cells gradually increases with the presence of HR-HPV positivity and an increasing CIN grade during the initial evaluation of women with or without abnormal cervical histology results. Moreover, an overexpression of PD-L1 is associated with the persistence of HR-HPV and the persistence or recurrence of CIN. During the initial evaluation of the cervical histology results, the assessment of PD-L1 expression—especially in mononuclear cells in cervical tissue samples—may provide more information on the progression of HR-HPV infection and its persistence. We suggest that a new scoring system consisting of PD-L1 immunoreactivity and histopathological findings be developed to estimate the clearance of the HR-HPV infection and the management of CIN lesions.

## Data Availability

The datasets generated during and/or analyzed during the current study are available from the corresponding author upon reasonable request.

## Abbreviations

PD-L1: Programmed cell death ligand 1
CIN: Cervical intraepithelial neoplasia
HPV: Human papilloma virus
SCC: Squamous cell carcinoma
Pap Test: Papanicolaou test
HR-HPV: High-risk HPV
ASCUS: Atypical squamous cells of undetermined significance
LSIL: Low-grade cervical intraepithelial lesion
HSIL: High-grade cervical intraepithelial lesion
ASC-H: Atypical squamous cells-cannot exclude high-grade squamous intraepithelial lesion
ANOVA: One-way analysis of variance
SD: Standard deviation
IHC: Immunohistochemistry
LEEP: Loop electrosurgical excision procedure
